# Radiation exposure to nuclear medicine technologists performing a V/Q PET: Comparison with conventional V/Q scintigraphy, [^18^F]FDG PET and [^68^Ga]Ga DOTATOC PET procedures

**DOI:** 10.3389/fmed.2022.1051249

**Published:** 2022-11-30

**Authors:** Frédérique Blanc-Béguin, Pascal Damien, Romain Floch, Kévin Kerleguer, Simon Hennebicq, Philippe Robin, Pierre-Yves Salaün, Pierre-Yves Le Roux

**Affiliations:** ^1^Medecine nucleaire, CHRU Brest, INSERM UMR 1304 (GETBO), Univ Brest, Brest, France; ^2^Unité de radioprotection et radiophysique, CHRU Brest, Brest, France; ^3^Medecine nucleaire, CHRU Brest, Brest, France

**Keywords:** radiation exposure, technologists, V/Q PET, V/Q scintigraphy, effective dose, finger dose

## Abstract

**Introduction:**

Ventilation/Perfusion (V/Q) PET/CT is an emerging imaging modality for regional lung function evaluation. The same carrier molecules as conventional V/Q scintigraphy are used but they are radiolabelled with gallium-68 (^68^Ga) instead of technetium-99m (^99m^Tc). A recurrent concern regarding V/Q PET imaging is the radiation dose to the healthcare workers. The aim of this study was to evaluate the total effective dose and the finger dose received by the technologist when performing a V/Q PET procedure, and to compare them with the radiations doses received with conventional V/Q scintigraphy, FDG PET and Ga DOTATOC PET procedures.

**Materials and methods:**

The whole body dose measurement was performed 10 times for each of the evaluated procedures using an electronic personal dosimeter (ED). For V/Q PET and V/Q scintigraphy procedures, ventilation and perfusion stages were separately evaluated. Internal exposure was measured for ventilation procedures. Finger dose measurements were performed 5 times for each of the PET procedures using Thermoluminescence (TL) pellets.

**Results:**

The technologist effective dose when performing a V/Q PET procedure was 2.83 ± 0.67 μSv, as compared with 1.16 ± 0.34 μSv for conventional V/Q scintigraphy, 2.13 ± 0.77 μSv for [^68^Ga]Ga-DOTATOC, and 2.86 ± 1.79 μSv for FDG PET procedures, respectively. The finger dose for the V/Q PET procedure was similar to the dose for a [^68^Ga]Ga-DOTATOC scan (0.35 mSv and 0.32 mSv, respectively).

**Conclusion:**

The technologist total effective dose for a V/Q PET procedure is ~2.4 higher than the dose for a conventional V/Q scintigraphy, but in the same range than the radiation exposure when performing common PET procedures, both in terms of total effective dose or finger dose. These results should be reassuring for the healthcare workers performing a V/Q PET procedure.

## Introduction

Ventilation/Perfusion (V/Q) PET is an emerging imaging modality for regional lung function evaluation (1). V/Q PET has demonstrated promising results in various pulmonary diseases, including pulmonary embolism diagnosis (2, 3) radiotherapy planning (4, 5) or preoperative evaluation for lung cancer surgery (6, 7). Several larger prospective clinical trials are also underway (NCT04179539, NCT03569072, NCT04942275, and NCT05103670).

The rationale of V/Q PET imaging is straightforward (8). The test uses the same carrier molecules as conventional V/Q scintigraphy, i.e., macroaggregated albumin (MAA) particles for perfusion imaging and aerosolized carbon nanoparticles for ventilation imaging. However, they are radiolabelled with gallium-68 (^68^Ga) instead of technetium-99m (^99m^Tc), allowing acquisition with PET, a vastly superior technology for image acquisition (9). Various manual or automated processes have been proposed to carry out [^68^Ga]Ga-MAA synthesis (10–14). For ventilation imaging, preparing and administering aerosolized ^68^Ga labeled carbon nanoparticles is very similar than with ^99m^Tc-labeled carbon nanoparticles. Indeed, adding a ^68^Ga eluate instead of ^99m^Tc eluate in the carbon crucible of an unmodified commercially available Technegas™ PLUS generator provides carbon nanoparticles with similar physical properties (15).

However, a recurrent concern regarding V/Q PET imaging is the radiation dose to the healthcare workers (16, 17). Indeed, positron-emitting radionuclides have high gamma photon energy (511 keV) as compared to ^99m^Tc radiopharmaceuticals (140 keV). Thus, the whole body dose measured when using PET radiopharmaceuticals is usually 2 to 4 times higher than with ^99m^Tc radiopharmaceuticals (18–21). This is of particular concern for ventilation PET imaging as the Technegas™ PLUS generator is designed to shield the 140-keV gamma-emissions of ^99m^Tc and not the 511-keV gamma-emissions of ^68^Ga. Given that ^68^Ga labeled carbon nanoparticles preparation requires operating the device during ~10 min, this may question the radiation safety of the technologist. Finally, when administrating ^68^Ga labeled carbon nanoparticles to the patient, the technologist may inhale aerosolized particles, leading to internal contamination.

The aim of this study was to evaluate the total effective dose and the finger dose received by the healthcare worker when performing a V/Q PET procedure, and to compare them with the radiations doses received with conventional V/Q scintigraphy, FDG PET and Ga DOTATOC PET procedures.

## Materials and methods

### Preparation and administration procedures

#### V/Q procedures

**Figure 1:**
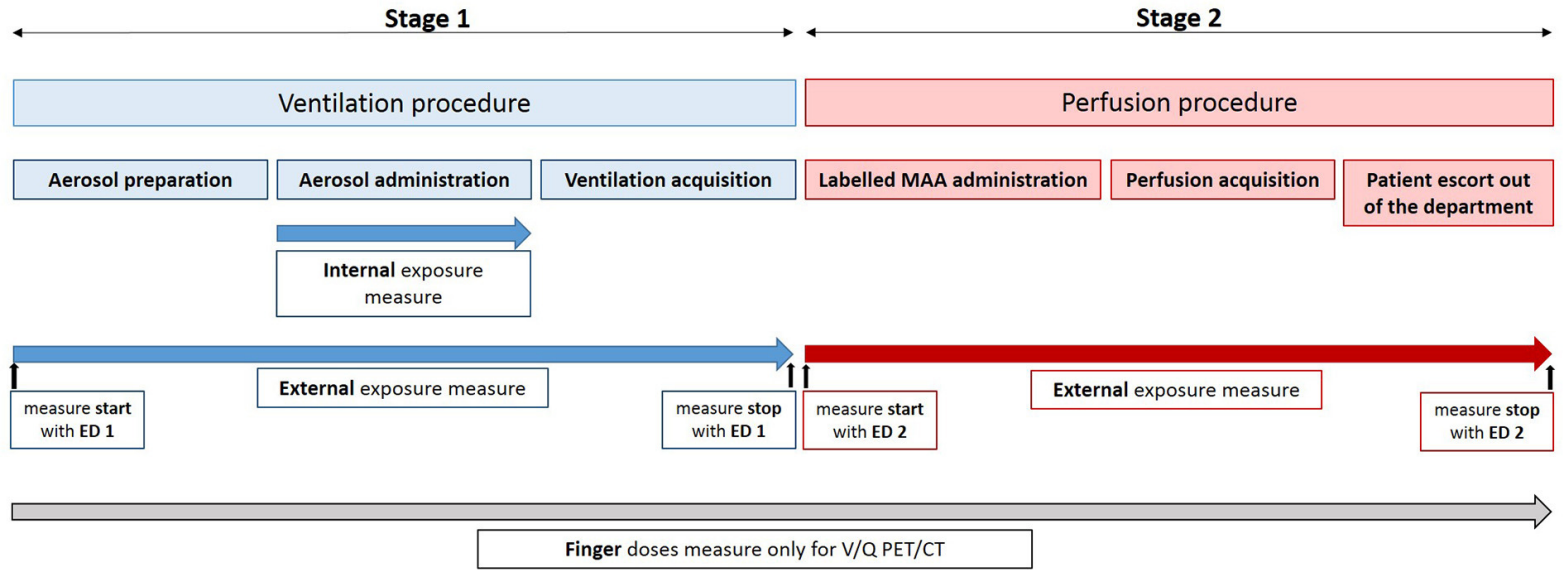
Summary of doses measurements for V/Q procedures: PET/CT and scintigraphy.

Both V/Q scintigraphy and PET procedures comprises two successive steps: the ventilation and the perfusion. The ventilation step can be broken down into three successive stages: the aerosol preparation, the aerosol administration, and the PET acquisition, respectively. It is immediately followed by the perfusion step which comprises labeled MAA administration, PET acquisition and patient escort out of the department after image acquisition. A summary of V/Q procedures and dose measurement is presented in Figure 1. All these stages were performed by nuclear medicine technologists.

##### V/Q PET

The aerosolized ^68^Ga-labeled carbon nanoparticles were prepared using an unmodified Technegas™ PLUS generator (Cyclomedica Pty Ltd., Australia), following the commonly used procedure (2, 8, 16, 17). Briefly, a ^68^Ga eluate (^68^GaCl_3_) was manually introduced in the graphite crucible previously humidified with 99% ethanol. After loading the crucible, the standard process used to prepare technegas was followed. The crucible was first heated for 6 min at 70°C during the simmer stage, and was then heated to 2,550 ± 50°C for 15 s in the high purity argon atmosphere during the burn stage. In order to increase the radioactivity contained in the aerosol, the crucible was loaded 2 times before the simmer stage.

The aerosol delivery was performed using the technegas administration system and a mask. Five to ten aerosol inhalation cycles were performed. The deposited activity in patient's lungs was monitored real-time during the aerosol administration by measuring the activity over the chest with a collimated Geiger-Müller monitor. During all the stage, the technologist was next to the patient. During the aerosol inhalation cycles, a gas venting system (LemerPax^®^, France) was used to capture the aerosol which could be released into the room.

Finally, the acquisition stage consisted in accompanying and positioning the patient on the PET/CT bed. The technologist did not stay near the patient during the acquisition time.

[^68^Ga]Ga-MAA suspension was prepared in the radiopharmacy using an automated process (miniAIO^®^, Trasis). This stage was performed by the radiopharmacist and was not included in the radiation dose measurement. [^68^Ga]Ga-MAA were manually administered by direct intravenous injection. The administration was done by the technologists while the patient was still lying down on the PET/CT bed, immediately after the ventilation PET acquisition. During the acquisition time, the technologists did not stay in the same room as the patient.

##### V/Q scintigraphy

The ^99m^Tc-labeled carbon nanoparticles (technegas) were prepared using a Technegas™ PLUS generator, following the instructions of the generator supplier. A ^99m^Tc eluate (^99m^${\text{TcO}}_{4}^{-}$, Na^+^) was manually introduced in the graphite crucible. Similar procedures as described for the aerosolized ^68^Ga-labeled carbon nanoparticles preparation and administration, and then image acquisition, were followed.

Similar to the perfusion PET/CT procedure, [^99m^Tc]Tc-MAA were prepared in the radiopharmacy. This stage was not included in the radiation dose measurement. [^99m^Tc]Tc-MAA were manually administered by the technologists by direct intravenous injection. The radiopharmaceuticals administration and the images acquisition were performed as previously described for the perfusion PET/CT procedure.

#### [^18^F]FDG and [^68^Ga]Ga-DOTATOC PET/CT procedures

[^18^F]FDG was a ready to use radiopharmaceutical purchased from CURIUM (Rennes, France). [^18^F]FDG was administered by the technologists with a semi-automated injector RAD-Inject^®^ (Tema Sinergie^®^, Italy).

The [^68^Ga]Ga-DOTATOC previously prepared in the radiopharmacy was manually intravenously administered by the technologists.

A [^18^F]FDG or [^68^Ga]Ga-DOTATOC procedures encompassed the time spent in administrating [^18^F]FDG or [^68^Ga]Ga-DOTATOC, escorting the patient to the PET room 1 h after the radiopharmaceutical administration, positioning the patient on the camera bed and escorting the patient out of the department after image acquisition.

#### Protection devices

All syringes were delivered in a tungsten syringe shield (Medisystem^®^, France) with wall thickness of 5 mm for ^68^Ga and ^18^F radiopharmaceuticals and 2 mm for ^99m^Tc radiopharmaceuticals, respectively. To transport syringes between the radiopharmacy, the labeled carbon nanoparticles preparation room, the administration room or the camera room, syringes were placed in tungsten carrying cases (LemerPax^®^, France) for gamma photon energy emitting radionuclides, with wall thickness of 10 mm for ^68^Ga and ^18^F radiopharmaceuticals and 3 mm thick for ^99m^Tc radiopharmaceuticals, respectively.

To introduce the eluate into the Technegas™ PLUS generator crucible and to administrate the labeled MAA to the patient, syringes were placed in tungsten shield with wall thickness of 5 mm for ^68^Ga radiopharmaceuticals and 2 mm for ^99m^Tc radiopharmaceuticals, respectively.

### Dose measurements

Radiation dose measurement was performed 10 times for each of the evaluated procedures, i.e., V/Q PET, V/Q scintigraphy, [^18^F]FDG PET scans and [^68^Ga]Ga-DOTATOC PET scans. Finger dose measurements were performed 5 times for each of the three PET procedures.

The whole body dose (= External exposure dose) was measured for all evaluated procedures using an electronic personal dosimeter (ED) EPD mk2 (APVL^®^, France) placed in the upper left pocket. For V/Q PET and V/Q scintigraphy procedures, ventilation and perfusion stages were separately evaluated. Technologists changed their ED between the ventilation and the perfusion phases (see Figure 1).

Because of the preparation and the administration of aerosolized labeled carbon nanoparticles, both internal and external exposures were assessed for the ventilation stages (see Figure 1).

Hence, in order to estimate the internal exposure dose of the technologist due to the inhalation of aerosolized ^99m^Tc- or ^68^Ga-labeled carbon nanoparticles, the inhaled dose was evaluated by collecting air sample from 5 min before to 5 min after the aerosol administration stage. The air was collected on a cellulose and glass fibers filter (collection efficiency up to 98%) with an aerosol collector PA 2000 (Algade^®^) at a flow of 33 L per minute. The filter radioactivity was measured immediately after the collection for 200 s with a gamma spectrometer equipped with a NaI (Tl) probe (Canberra^®^) and calibrated for ^99m^Tc and ^68^Ga.

The finger doses were measured using Thermoluminescence (TL) pellets worn on both hands thumb and index finger tip during all the procedures (see Figure 1). The received dose by the irradiated TL pellets was determined by the manufacturer (IRSN^®^, France). The worst case-scenario was retained.

### Dose calculation

Calculation of the effective doses was performed as follows according to IRSN guideline (report PRP-HOM/DIR n°2015-00009).

The technologist total effective dose of the V/Q procedures was calculated as follows:


$$\begin{array}{l} {\text{Total E = E}}_{\text{(ventilation internal exposure dose)}}\\ {\text{ + E}}_{\text{(ventilation external exposure dose)}}\\ {\text{ + E}}_{\text{(perfusion external exposuredose)}} \end{array}$$

Where E was the effective dose expressed in mSv.

HP(10) value obtained from the ED was used as the dose due to external exposure. The mean effective dose for each procedures was used.

The effective dose due to ventilation internal exposure was calculated as follows:


$$\begin{array}{l} {\text{E}}_{\left(\text{ventilation internal exposure dose}\right)}=\text{I }\times \text{ h}\left(\text{g}\right) \end{array}$$

Where I is the Incorporation expressed in Bq,

h(g) is the effective dose per intake by inhalation (μSv.Bq^−1^). For ^68^Ga, h(g) = 2.8 x 10^−5^ μSv.Bq^−1^, for ^99m^Tc, h(g) = 1.2 × 10^−5^.

I was evaluated as follows by assuming that all the inhaled air remained in the lungs:


$$\begin{array}{l} \text{I}=\text{d }\times \text{ t }\times \text{ c} \end{array}$$

Where: d = operator respiratory flow = 1.2 m^3^/h,

t = measurement time = 10 min,

c = ^68^Ga or ^99m^Tc concentration in the air (Bq/m^3^).

The exposure doses results and time results were expressed as mean ± SD. The Mann-Whitney test was performed to statistically compare total effective doses and finger doses.

## Results

Table 1 shows results of effective dose measurement of ^68^Ga V/Q PET and ^99m^Tc V/Q scintigraphy procedures. Table 2 shows technologists total effective dose and finger doses of ^68^Ga V/Q PET, ^18^F FDG PET and ^68^Ga V/Q PET procedures.

**Table 1:** Results of effective doses due to pulmonary ventilation and perfusion, and total effective dose according to the radionuclide used to performed V/Q exam.

		**Ventilation**			**Perfusion**	**Total**
		**External exposure dose (μSv)**	**Internal exposure dose (μSv)**	**Total exposure dose (μSv)**	**Total exposure dose (μSv)**	**Total effective dose (μSv)**
V/Q PET	Mean	1.71	0.22	1.93	0.90	2.83
	SD	0.48	0.31	0.60	0.30	0.67
V/Q scintigraphy	Mean	0.67	0.08	0.75	0.41	1.16
	SD	0.26	0.09	0.25	0.24	0.34

**Table 2:** Technologists total effective dose and finger doses according to the exam performed, total process timing, and syringes mean activity.

	**Total effective dose (μSv)**	**Finger dose (mSv)**	**Process mean duration (min)**	**Syringes mean activity (MBq)**
[^68^Ga]Ga-DOTATOC	2.13 ± 0.77	0.32	66 ± 7	207.81 ± 52.80
[^18^F]FDG	2.86 ± 1.79	0.01	75 ± 9	231.88 ± 1.79.80
V/Q PET/CT	2.83 ± 0.67	0.35	68 ± 20	105.55 ± 8.59

[^18^F]FDG was injected with an automated injector.

### V/Q PET

Mean activity of ^68^Ga eluate used to prepare labeled carbon nanoparticles was 56.30 ± 8.42 MBq. Mean activity of [^68^Ga]Ga-MAA contained in the syringes was 49.24 ± 7.22 MBq.

The total exposure dose for the ventilation and the perfusion procedures was 1.93 ± 0.60 μSv and 0.9 ± 0.3 μSv, respectively (Table 1). The total effective dose of the V/Q PET procedure was 2.83 ± 2.67 μSv. The internal exposure was 0.22 ± 0.31 μSv. The finger dose obtained in the worst case-scenario was 0.35 mSv (Table 2).

### V/Q scintigraphy

Mean activity of ^99m^Tc eluate used to prepare labeled carbon nanoparticles was 582.0 ± 71.1 MBq. Mean activity of [^99m^Tc]Tc-MAA contained in the syringes was 170.9 ± 12.9 MBq.

The total exposure dose for the ventilation and perfusion procedures was 0.75 ± 0.25 μSv and 0.41 ± 0.24 μSv, respectively. The total effective dose for V/Q scintigraphy was 1.16 ± 0.34 μSv, significantly lower as compared with the total effective dose of the V/Q PET Procedure (*p* < 0.001). The internal exposure was 0.08 ± 0.09 μSv.

### [^18^F]FDG process

The mean dose administered for a [^18^F]FDG procedure was 231.88 ± 70.91 MBq. Mean total effective dose of the [^18^F]FDG PET process was 2.86 ± 1.79 μSv.

There was no significant difference between the total effective doses of the V/Q PET and the [^18^F]FDG PET procedures (*p* = *0.307*). The finger dose obtained in the worst case-scenario was 0.01 mSv.

### [^68^Ga]Ga-DOTATOC process

The mean dose administered to the patient of 207.81 ± 52.80 MBq (Table 2). The total effective dose of the [^68^Ga]Ga-DOTATOC procedure was 2.13 ± 0.77 μSv, without significant difference as compared with the V/Q PET procedure (*p* = 0,043). The finger dose obtained in the worst case-scenario was 0.32 mSv.

## Discussion

To the best of our knowledge, this is the first study that assessed the radiation exposure to the healthcare worker when performing a V/Q PET procedure. We found that the total effective dose for a V/Q PET procedure was about 2.4 times higher than the dose for a V/Q scintigraphy, but in the same range than the radiation exposure due to a [^68^Ga]Ga-DOTATOC or [^18^F]FDG PET procedure.

In our study, the technologist effective dose when performing a V/Q PET procedure was 2.83 ± 0.67 μSv, as compared with 1.16 ± 0.34 μSv for a conventional V/Q scintigraphy procedure. Measured doses in this study for conventional V/Q scintigraphy are consistent with literature data. Indeed, whole body mean dose due to perfusion lung scan with [^99m^Tc]Tc-MAA was reported between 0.4 ± 0.2 μSv and 1.1 ± 1.2 μSv, as compared with 0.41 ± 0.24 μSv in our study (19, 21). Few data about the internal dose due to ventilation lung scan are available in the literature and values are very different from one work to another. Indeed, internal exposures of 0.002 μSv and 0.17 μSv per ventilation lung scan were reported (22, 23). The internal exposure of 0.08 μSv per ventilation lung scan found in this study was in the range of this data.

The effective dose for a V/Q PET procedure was 2.4 times higher than for a V/Q scintigraphy. This is also consistent with literature data which mentioned a whole body dose from 2 to 4 times higher with positron-emitting radionuclides than with radionuclides for conventional scintigraphy (18–21). As compared with other PET procedures, the radiation dose for the V/Q PET was of particular concern given that the ^68^Ga labeled carbon nanoparticles preparation requires operating the device during ~10 min and that the Technegas™ PLUS generator is not designed to shield the 511-keV gamma-emissions of ^68^Ga. Furthermore, the technologist inhale aerosolized particles leading to internal contamination. However, we found that without adding a lead shield to the Technegas™ PLUS generator, the percentage of the total effective dose due to the ventilation procedure was in the same range for V/Q PET/CT and V/Q scintigraphy (68 and 65%, respectively). It is worth noting that the higher energy of PET tracers is partly counter-balanced by the lower activity handled for the V/Q PET procedure: 56.30 ± 8.42 MBq of ^68^Ga eluate vs. 582.0 ± 71.1 MBq of ^99m^Tc eluate for the carbon nanoparticles labeling, and 49.24 ± 7.22 MBq of [^68^Ga]Ga-MAA vs 170.9 ± 12.9 MBq for the perfusion, respectively.

The radiation dose of the V/Q PET procedure should be put into perspective with commonly used PET tracers. The total effective dose of a V/Q PET (2.83 ± 0.67 μSv) was in the same range than the dose calculated for other procedures performed with high photon gamma energy emitters. Indeed, the total effective dose for a [^68^Ga]Ga-DOTATOC and a FDG procedure was 2.13 ± 0.77 μSv and 2.86 ± 1.79 μSv, respectively.

For [^18^F]FDG procedures, total effective dose ranging from 2.5 to 2.9 μSv were reported (20).

The finger dose for the V/Q PET procedure was also similar to the dose for a [^68^Ga]Ga-DOTATOC scan (0.35 and 0.32 mSv, respectively). These measured doses were lower than the finger doses previously published by Diwedi et al. for [^68^Ga]Ga-DOTANOC injection, which were 1.26 ± 0.3 mSv for the left ring finger and 1.03 ± 0.13 mSv for right ring finger (24). In this study, the same range of activity was injected, but no syringe shield was used in contrast to the present work. In our study, the total of radioactivity handled by technologists for the V/Q PET procedure was two times less than the syringe activity for [^68^Ga]Ga-DOTATOC (105.55 ± 8.59 MBq and 207.81 ± 52.80 MBq, respectively). This was likely counter-balanced by a longer and more complicated handling of the syringes for the V/Q PET procedure. In both V/Q PET and DOTATOC procedures, the syringes were manually administered. In contrast, [^18^F]FDG was administrated with automated injector, which explains the lower finger dose for the FDG PET procedure (0.01 mSv).

Our results should also be put into perspective with the technical advantage of V/Q PET over conventional V/Q SPECT scintigraphy. Indeed, according to the ALARA principle and given that the radiation exposure is higher with V/Q PET than with V/Q SPECT imaging, V/Q PET imaging should only be used when the test provides higher diagnostic performance. V/Q PET/CT is inherently a superior technology for image acquisition, with higher sensitivity and spatial resolution, allowing more accurate evaluation of regional lung function (1). Other advantages include reduced acquisition time and the opportunity to readily perform respiratory-gated acquisition (25). Preliminary results showed promising results for the diagnosis of acute pulmonary embolism (1, 2), pending for the results of ongoing diagnostic accuracy studies (NCT04179539). Another clinical indication for which PET has an additional value over SPECT imaging is radiotherapy planning, for individualizing treatment plans and predicting radiation induced lung injury (4, 5, 7, 26–29).

Limitations have to be considered in this work. First, the size of the samples was small (10 measures for each investigated procedures). Second, measures for the V/Q PET procedures were performed at the start of the use of this new technology in our department. Although the process is very similar as compared with technegas preparation, the radiation exposure measures may have been overestimated due to an increased duration of the procedures. On the other hand, measures in this study have been obtained in the worst case-scenario, which is often retained for radiation exposure calculation. Third, the perfusion total exposure dose of the V/Q PET procedure include some radiation from the ^68^Ga-labeled carbon nanoparticles previously inhaled by the patient. In some clinical setting, such as radiotherapy planning (5, 25), only a perfusion PET/CT scan (without ventilation) is performed. In that context, the total effective dose for a perfusion PET procedure would likely be lower than that reported in this work.

Fourth, we cannot assert that the collection of air samples is an exact representation of aerosolized particles inhaled by the technologist. However, the use of an aerosol collector is a common method for air contamination studies (22, 23, 30). Most importantly, similar collection method was used for Tc and Ga aerosols, allowing the comparison between both procedures.

## Conclusion

The technologist total effective dose for a V/Q PET procedure is ~2.4 higher than the dose for a conventional V/Q scintigraphy procedure. However, the radiation exposure for a V/Q PET procedure is equivalent to the exposure of other PET procedures, both in terms of total effective dose or finger dose. These results should be reassuring for the healthcare workers performing V/Q PET scan.

## Data availability statement

The original contributions presented in the study are included in the article/supplementary material, further inquiries can be directed to the corresponding authors.

## Author contributions

Dose measurements were performed by FB-B, RF, KK, and SH. Dose calculations were performed by PD and FB-B. The first draft of the manuscript was written by FB-B and P-YL. All authors commented on previous versions of the manuscript, contributed to the study conception and design, and read and approved the final manuscript.

## Conflict of interest

The authors declare that the research was conducted in the absence of any commercial or financial relationships that could be construed as a potential conflict of interest.

## Publisher's note

All claims expressed in this article are solely those of the authors and do not necessarily represent those of their affiliated organizations, or those of the publisher, the editors and the reviewers. Any product that may be evaluated in this article, or claim that may be made by its manufacturer, is not guaranteed or endorsed by the publisher.

## References

[1] Le Roux PY Hicks RJ Siva S Hofman MS. PET/CT Lung ventilation and perfusion scanning using galligas and gallium-68-MAA. Semin Nuclear Med. (2019) 49:71–81. 10.1053/j.semnuclmed.2018.10.013 30545520

[2] Hofman MS Beauregard JM Barber TW Neels OC Eu P Hicks RJ. 68Ga PET/CT ventilation-perfusion imaging for pulmonary embolism: a pilot study with comparison to conventional scintigraphy. J Nuclear Med. (2011) 52:1513–9. 10.2967/jnumed.111.093344 21908388

[3] Le Roux P-Y Iravani A Callahan J Burbury K Eu P Steinfort DP. Independent and incremental value of ventilation/perfusion PET/CT and CT pulmonary angiography for pulmonary embolism diagnosis: results of the PECAN pilot study. Eur J Nucl Med Mol Imaging. (2019) 46:1596–604. 10.1007/s00259-019-04338-z 31044265

[4] Siva S Thomas R Callahan J Hardcastle N Pham D Kron T. High-resolution pulmonary ventilation and perfusion PET/CT allows for functionally adapted intensity modulated radiotherapy in lung cancer. Radiother Oncol. (2015) 115:157–62. 10.1016/j.radonc.2015.04.013 25935743

[5] Lucia F Rehn M Blanc-Beguin F Le Roux PY. radiation therapy planning of thoracic tumors: a review of challenges associated with lung toxicities and potential perspectives of gallium-68 lung PET/CT imaging. Front Med. (2021) 8:723748. 10.3389/fmed.2021.723748 34513884 PMC8429617

[6] Le Roux PY Siva S Steinfort DP Callahan J Eu P Irving LB. Correlation of 68Ga ventilation-perfusion PET/CT with pulmonary function test indices for assessing lung function. J Nuclear Med. (2015) 56:1718–23. 10.2967/jnumed.115.162586 26338892

[7] Le Roux PY Leong TL Barnett SA Hicks RJ Callahan J Eu P. Gallium-68 perfusion positron emission tomography/computed tomography to assess pulmonary function in lung cancer patients undergoing surgery. Cancer Imaging. (2016) 16:24. 10.1186/s40644-016-0081-5 27544383 PMC4992565

[8] Blanc-Beguin F Hennebicq S Robin P Tripier R Salaun PY Le Roux PY. Radiopharmaceutical labelling for lung ventilation/perfusion PET/CT imaging: a review of production and optimization processes for clinical use. Pharmaceuticals. (2022) 15:518. 10.3390/ph15050518 35631345 PMC9143102

[9] Hicks RJ Hofman MS. Is there still a role for SPECT-CT in oncology in the PET-CT era? Nat Rev Clin Oncol. (2012) 9:712–20. 10.1038/nrclinonc.2012.188 23149896

[10] Blanc-Béguin F Masset J Robin P Tripier R Hennebicq H Guilloux V. Fully automated ^68^Ga-labeling and purification of macroaggregated albumin particles for lung perfusion PET imaging. Front Nuclear Med. (2021) 1:10. 10.3389/fnume.2021.778191 PMC11440869 39355632

[11] Mueller D Kulkarni H Baum RP Odparlik A. Rapid Synthesis of (68)Ga-labeled macroaggregated human serum albumin (MAA) for routine application in perfusion imaging using PET/CT. Appl Radiat Isot. (2017) 122:72–7. 10.1016/j.apradiso.2017.01.003 28113072

[12] Maus S Buchholz HG Ament S Brochhausen C Bausbacher N Schreckenberger M. Labelling of commercially available human serum albumin kits with 68Ga as surrogates for 99mTc-MAA microspheres. Appl Radiat Isot. (2011) 69:171–5. 10.1016/j.apradiso.2010.09.008 20880714

[13] Even GA Green MA. Gallium-68-labeled macroaggregated human serum albumin, 68Ga-MAA. Int J Radiat Applic Instrumenta Part B Nuclear Med Biol. (1989) 16:319–21. 10.1016/0883-2897(89)90014-7 2785514

[14] Jain A Subramanian S Pandey U Sarma HD Ram R Dash A. In-house preparation of macroaggregated albumin (MAA) for ^68^Ga labeling and its comparison with comercially available MAA. J radioanal Nucl Chem. (2016) 308:8. 10.1007/s10967-015-4509-3

[15] Blanc-Beguin F Elies P Robin P Tripier R Kervarec N Lemarie CA. (68)Ga-labelled carbon nanoparticles for ventilation PET/CT imaging: physical properties study and comparison with technegas(R). Mol Imaging Biol. (2021) 23:62–9. 10.1007/s11307-020-01532-6 32886302

[16] Currie GM Bailey DL. A technical overview of technegas as a lung ventilation agent. J Nucl Med Technol. (2021) 49:313–9. 10.2967/jnmt.121.262887 34583954

[17] Bailey DL Eslick EM Schembri GP Roach PJ. (68)Ga PET ventilation and perfusion lung imaging-current status and future challenges. Semin Nuclear Med. (2016) 46:428–35. 10.1053/j.semnuclmed.2016.04.007 27553468

[18] Benatar NA Cronin BF O'Doherty MJ. Radiation dose rates from patients undergoing PET: implications for technologists and waiting areas. Eur J Nuclear Med. (2000) 27:583–9. 10.1007/s002590050546 10853815

[19] Chiesa C De Sanctis V Crippa F Schiavini M Fraigola CE Bogni A. Radiation dose to technicians per nuclear medicine procedure: comparison between technetium-99m, gallium-67, and iodine-131 radiotracers and fluorine-18 fluorodeoxyglucose. Eur J Nucl Med. (1997) 24:1380–9. 10.1007/s002590050164 9371871

[20] Guillet B Quentin P Waultier S Bourrelly M Pisano P Mundler O. Technologist radiation exposure in routine clinical practice with 18F-FDG PET. J Nuclear Med Technol. (2005) 33:175–9. 16145226

[21] Sloboda RS Schmid MG Willis CP. Technologist radiation exposures from nuclear medicine imaging procedures. J Nuclear Med Technol. (1987) 15:8.

[22] Ferrand O Brouquières G Puech B Bussy E. Zonage radiologique d'un service de médecine nucléaire : exemple de l'hôpital d'instruction des armées Sainte-Anne. Méd Nucléaire. (2010) 34:11. 10.1016/j.mednuc.2010.10.006

[23] Brudecki K Borkowska E Gorzkiewicz K Kostkiewicz M Mroz T. (99m)Tc activity concentrations in room air and resulting internal contamination of medical personnel during ventilation-perfusion lung scans. Radiat Environ Biophys. (2019) 58:469–75. 10.1007/s00411-019-00793-2 30997611 PMC6609588

[24] Dwivedi DK Snehlata Dwivedi AK Lochab SP Kumar R Naswa N. Radiation exposure to nuclear medicine personnel handling positron emitters from Ge-68/Ga-68 generator. Indian journal of nuclear medicine : *IJNM*. (2011) 26:86–90. 10.4103/0972-3919.90258 22174513 PMC3237224

[25] Le Roux PY Siva S Callahan J Claudic Y Bourhis D Steinfort DP. Automatic delineation of functional lung volumes with (68)Ga-ventilation/perfusion PET/CT. EJNMMI Res. (2017) 7:82. 10.1186/s13550-017-0332-x 29019109 PMC5634989

[26] Hardcastle N Hofman MS Hicks RJ Callahan J Kron T MacManus MP. Accuracy and utility of deformable image registration in (68)Ga 4D PET/CT assessment of pulmonary perfusion changes during and after lung radiation therapy. Int J Radiat Oncol Biol Phys. (2015) 93:196–204. 10.1016/j.ijrobp.2015.05.011 26279034

[27] Siva S Hardcastle N Kron T Bressel M Callahan J MacManus MP. Ventilation/perfusion positron emission tomography–based assessment of radiation injury to lung. Int J Radiat Oncol Biol Phys. (2015) 93:408–17. 10.1016/j.ijrobp.2015.06.005 26275510

[28] Argula RG Strange C Ramakrishnan V Goldin J. Baseline regional perfusion impacts exercise response to endobronchial valve therapy in advanced pulmonary emphysema. Chest. (2013) 144:1578–86. 10.1378/chest.12-2826 23828481

[29] Leong P Le Roux PY Callahan J Siva S Hofman MS Steinfort DP. Reduced ventilation-perfusion (V/Q) mismatch following endobronchial valve insertion demonstrated by Gallium-68 V/Q photon emission tomography/computed tomography. Respirol Case Rep. (2017) 5:e00253. 10.1002/rcr2.253 28808575 PMC5550810

[30] Martinez J Baciu T Artigues M Danus M Penalver A Aguilar C. Nuclear medicine: workplace monitoring and internal occupational exposure during a ventilation/perfusion single-photon emission tomography. Radiat Environ Biophys. (2019) 58:407–15. 10.1007/s00411-019-00798-x 31139897

